# Investigating the Relationship Between Midazolam Serum Concentrations and Paediatric Delirium in Critically Ill Children

**DOI:** 10.3390/pediatric17010007

**Published:** 2025-01-14

**Authors:** Sabrina Marongiu, Mathieu S. Bolhuis, Daan J. Touw, Martin C. J. Kneyber

**Affiliations:** 1 Department of Clinical Pharmacy and Pharmacology, University Medical Center Groningen, University of Groningen, 9713 GZ Groningen, The Netherlands; m.s.bolhuis@umcg.nl (M.S.B.); d.j.touw@umcg.nl (D.J.T.); 2 Department of Pharmaceuticals Analysis, Groningen Research Institute of Pharmacy (GRIP), University of Groningen, 9713 AV Groningen, The Netherlands; 3 Department of Pediatrics, Division of Pediatric Critical Care Medicine, University Medical Center Groningen, University of Groningen, 9713 GZ Groningen, The Netherlands; m.c.j.kneyber@umcg.nl; 4 Critical Care, Anaesthesiology, Peri–Operative & Emergency Medicine (CAPE), University Medical Center Groningen, University of Groningen, 9713 GZ Groningen, The Netherlands

**Keywords:** midazolam, delirium, paediatric intensive care, pharmacology

## Abstract

Objectives: Intravenous midazolam is widely used for sedation in critically ill children. Sometimes, these children develop a paediatric delirium (PD). Our aim was to determine the relationship between midazolam serum concentration and the development of new PD in critically ill children. Design: Prospective observational pilot study. Setting: Paediatric Intensive Care Unit (PICU), Groningen, the Netherlands. Patients: All children admitted to the PICU from October–December 2019 who received continuous midazolam administration. Interventions: None. Measurements and main results: Twenty-five percent (n = 7) of the included patients (n = 28) developed new PD. In most patients, PD occurred following midazolam dose reduction. The median cumulative midazolam dose was higher in patients who developed PD compared to those without PD. We analysed 104 blood samples to determine the midazolam concentrations. To determine whether patients had PD, the Sophia Observation withdrawal Symptoms-Paediatric Delirium (SOS-PD) score was used. Patients suffering PD (n = 7) had a lower median midazolam concentration on that day compared with the day prior to PD detection. Analysis of the active metabolites, 1-hydroxymidazolam and 1-hydroxymidazolam glucuronide, showed similar results. Conclusions: PD may be linked to a sudden and significant reduction in the midazolam concentration in critically ill children. Further investigation in larger patient populations is necessary to validate our findings.

## 1. Introduction

### 1.1. Paediatric Delirium

Developing paediatric delirium (PD) in children in the Paediatric Intensive Care Unit (PICU) occurs in up to 47% of critically ill children [1]. PD is an acute neurological dysfunction involving impairment of consciousness, attention, and/or cognition [2]. A PD has a fluctuating course and may be accompanied by anxiety, disorientation, confusion, decreased eye contact, motor agitation, apathy, hallucinations, and disturbed sleep patterns [3,4]. The pathophysiology of a PD is complex and multifactorial [5]. The underlying mechanism relies on disruption of neurotransmitter function and cellular homeostasis, increased energy metabolism, and decreased cerebral blood flow [3,6]. A PD is associated with longer hospitalization time, increased morbidity, and mortality [3,7].

### 1.2. Midazolam in the PICU

The benzodiazepine midazolam is widely used in mechanically ventilated children [8]. Midazolam is used in the PICU because of its sedative, anxiolytic, muscle relaxant, and anticonvulsant effect [9]. In our clinic, midazolam is administered via continuous midazolam administration (dosage range 0.1–0.4 mg/kg/h) and titrated by clinical response and COMFORT Behaviour scale [10]. In addition to these desirable properties of midazolam in the PICU, intravenous midazolam may induce paradoxical reactions in children, such as hallucination, disorientation, agitation, restlessness, or violent behaviour [11]. Furthermore, continuous midazolam administration is increasingly being identified as an independent PD risk factor [8].

### 1.3. Pharmacokinetics and Interactions

For ventilated patients in the PICU, midazolam is one of the most widely used sedatives due to its relatively short half-life [9]. The short half-life of midazolam is desirable because after extubation, the sedative and muscle relaxant effect may be minimal. Midazolam is metabolised in the liver by cytochrome P450 (CYP) enzymes to the primary active metabolite 1-hydroxymidazolam, followed by 1-hydroxymidazolam glucuronide after glucuronidation, which is renally excreted [12]. The active metabolites 1-hydroxymidazolam and 1-hydroxymidazolam glucuronide have sedative potencies of 80% and 10%, respectively, compared to midazolam [13,14,15]. The half-life of midazolam has a large interindividual variability due to factors such as age and severity of illness [9]. The half-life of midazolam in healthy children and adults is 1.5–3 h and 1 h of its metabolite 1-hydroxymidazolam [16]. The midazolam half-life in neonates is delayed, between 4 and 6 h, because CYP3A4 activity is not yet fully developed [9].

In clinical practice, several factors may influence midazolam pharmacokinetics. Differences in hepatic blood flow, through shock and multi-organ failure, affect the clearance of midazolam [17]. Critically ill patients with impaired liver function could be more strongly sedated by midazolam than patients without hepatic impairment [18]. In addition, the administration of multiple drugs may affect the midazolam clearance in patients due to CYP3A4 interactions [17]. Fluconazole is a commonly used antimycotic agent in the PICU and may cause an increase in midazolam level due to CYP3A4 inhibition [19]. Phenobarbital is a CYP3A4 inducer and therefore may cause increased midazolam clearance, resulting in a lower midazolam concentration [20]. Patients with renal failure may experience a stronger effect of midazolam due to reduced renal clearance and a reduction in albumin, resulting in an increase in free active midazolam concentration [17]. In addition, the sedative effect of midazolam may be prolonged due to accumulation of 1-hydroxymidazolam glucuronide [12,14].

### 1.4. Identification of PD

The most common cause of PD in the PICU is severe illness [3]. Other risk factors include mechanical ventilation, the presence of intravenous lines and an unfamiliar environment, or the administration of psychoactive drugs such as benzodiazepines [5,21,22]. Identifying a PD in patients in the PICU is difficult because symptoms overlap with symptoms of children with severe pain, sedation, and withdrawal symptoms [4,23]. Epidemiology and risk factors of PD have been sparsely described so far, due to insufficient available data and few validated measurement tools [24,25].

### 1.5. Objectives and Hypothesis

This study aimed to investigate whether daily changes in midazolam serum concentration were associated with the development of new PD in critically ill children receiving continuous midazolam in the PICU (primary objective). A secondary objective was to evaluate whether the midazolam dosage influenced PD onset. We hypothesised that higher serum concentrations of midazolam, as well as increased dosages, correlated with an elevated risk of developing PD in this patient population.

## 2. Materials and Methods

### 2.1. Design and Setting

The pilot study was designed as a single-centre, prospective, observational study conducted in the PICU of UMCG. Patients were admitted to the PICU between October and December 2019. The need for informed consent was waived by the Institutional Review Board (IRB UMCG, METc 2019/657; UMCG RR 201900655).

### 2.2. Inclusion and Exclusion Criteria

Inclusion criteria included patients aged 0–18 years for whom a Sophia Observation withdrawal Symptoms-Paediatric Delirium (SOS-PD) score screening was performed, patients > 48 h admitted to the PICU, patients on continuous midazolam infusion (whether or not combined with boluses), and patients whose blood gas samples were routinely collected via an arterial line. The children admitted to the PICU presented with diverse reasons for admission, including congenital heart disease, liver or kidney transplantation, and respiratory insufficiency. Exclusion criteria were deeply sedated patients in whom SOS-PD screening was not possible. Because this was an explorative study, there was no requirement for population size. The goal was to include all patients who met the inclusion criteria during this period. Each morning (Monday–Friday), we identified new PICU admissions meeting inclusion criteria. Over the weekend, nurses collected blood samples from all new admissions, which were reviewed on Monday to confirm eligibility.

### 2.3. Data Collection

#### 2.3.1. Midazolam Concentrations

For the primary endpoint, plasma concentrations of midazolam and its active metabolites, 1-hydroxymidazolam and 1-hydroxymidazolam glucuronide, were measured in left-over material of routinely drawn blood samples using an in-house developed and validated LC-MS/MS method. After serum–heparin cross-validation, human heparin plasma samples were allowed to be calculated on a two-point calibration in human serum. The analytical method of midazolam, 1-hydroxymidazolam, and 1-hydroxymidazolam glucuronide was performed using TSQ Quantiva LC-MS/MS and the internal standards cyanoimipramine and [13C6]-1-hydroxymidazolam glucuronide. For midazolam and 1-hydroxymidazolam, a two-point calibration with 5 and 1500 mcg/L and for 1-hydroxymidazolam glucuronide with 25 and 5000 mcg/L was used. The method was validated according to FDA and EMA regulations and validation parameters complied with the requirements.

We compared the median (interquartile range, IQR) percentage reduction values of the midazolam concentration between the PD and non-PD group. For the PD group, we calculated the percentage reduction in midazolam concentration on the day with PD compared to the day prior to PD. For the non-PD group, we used two consecutive days, which had the strongest positive or negative change in midazolam concentration during their PICU admission.

For pharmacological descriptions including midazolam metabolites, midazolam metabolites were multiplied by the potency. Serum concentration midazolam equivalents = (1 × midazolam) + (0.8 × 1-hydroxymidazolam) + (0.1 × 1-hydroxymidazolam glucuronide).

#### 2.3.2. Midazolam Dosage

For the secondary endpoint, midazolam doses were collected via the Electronic Health Record (EHR) using the program Epic Systems Corporation (Madison, WI, USA, 2019). The median cumulative midazolam dose during PICU admission was calculated. In addition, the daily midazolam doses were determined per patient.

#### 2.3.3. SOS-PD Score

Screening for PD using the validated tool, SOS-PD score, was routinely executed in our unit [26]. Children admitted to the PICU for more than 48 h were screened three times a day, once each shift, by a nurse as part of the routine measurement of discomfort. All nurses were trained in applying the SOS-PD score through a structured program. This included theoretical instruction, followed by the application of the PD scale while watching video material of three cases of PD diagnosed by a child psychiatrist. Nurses’ scores were compared with a reference score provided by the instructor, and discrepancies were explained, with additional advice for observation in clinical practice. First-tier treatment of patients with psychiatrist-confirmed PD (SOS-PD ≥ 4) included non-pharmacological interventions. Second-tier treatment included prescription of risperidone.

#### 2.3.4. Patient Characteristics

To collect the baseline demographical and clinical characteristics, the EHR was accessed. The following values were collected: gender, age at admission, weight, height, Body Mass Index (BMI), admission diagnoses, Paediatric Risk of Mortality (PRISM III) 24 hr score, renal function determined using the paediatric-Risk Injury Failure Loss End stage (p-RIFLE) score and need for dialysis, the type and length of respiratory support, PICU length of stay, previous hospitalization, and PICU survival [27,28].

#### 2.3.5. Co-Medication

Concomitant use of other relevant medications with continuous midazolam administration were selected based on interaction potential and psychoactive properties. Medication involved fentanyl, morphine, lorazepam, phenobarbital, and fluconazole. The dosages were retrieved from the EHR.

#### 2.3.6. Statistical Analyses

The program RStudio, version 3.6.2, Boston, MA, USA, was used for the statistical analyses. For unpaired data, a Fisher’s exact test was used, and for continuous data, a Mann–Whitney U test was used. For continuous paired data, a Wilcoxon signed rank test was used. All data were presented using the median (25–75 IQR). *p*-values < 0.05 were considered statistically significant.

## 3. Results

We analysed 104 convenience blood samples of midazolam concentration with a linked SOS-PD score in 28 patients (Table 1). Seven (25%) patients developed a PD during PICU admission. The median (IQR) time between PICU admission and detection of PD was 8 (6.3–28.5) days. Baseline demographical and clinical characteristics between patients with PD or patients without PD were comparable (Table 1). During the PICU admission, patients received several drugs that potentially interact with midazolam. There was no difference in use of fentanyl, morphine, lorazepam, phenobarbital, and fluconazole between patients with and without PD (Table 2).

**Figure 1:**
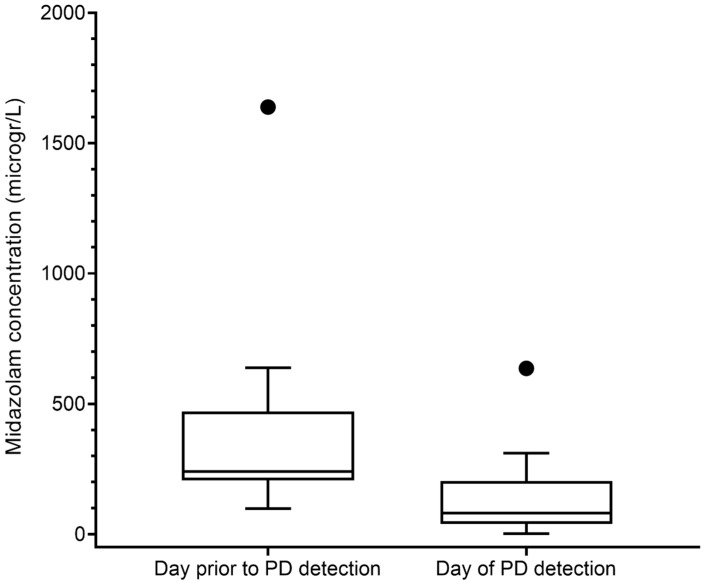
Midazolam concentration (µg /L) in patients with paediatric delirium (PD) at that time point and the day prior to PD. Data are expressed as median (25–75 interquartile range, IQR).

The median midazolam concentration was significantly lower in samples taken from patients during PD (80.9 [38.7–204.3] µg/L, n = 7) compared to samples taken on a day without PD (428.4 [222.5–889.3] µg/L, n = 97, *p* = 0.004). Furthermore, if we focus on the patients with PD, patients with PD had a significantly lower median midazolam concentration at the time point they had PD (80.9 [38.7–204.3] µg/L) compared with the day prior to PD detection (239.7 [206.4–470.2] µg/L, n = 7, *p* = 0.031) (Figure 1). When the concentrations of midazolam, including its active metabolites 1-hydroxymidazolam and 1-hydroxymidazolam glucuronide, were considered, patients also had a significantly lower concentration on the day they had PD (157.0 [98.2–355.9] µg/L) compared to the day prior to PD (390.4 [334.2–597.2] µg/L, n = 7, *p* = 0.028).

The median cumulative midazolam dose during PICU admission was significantly higher in patients with PD (30.7 [21.1–37.1] mg/kg, n = 7) than those without PD (13.6 [11.8–26.6] mg/kg, n = 21, *p* = 0.036). Focusing on patients with PD, the median midazolam dose was significantly lower on the day of PD (0.9 [0.1–1.0] mg/kg/day) compared to the day prior PD (3.5 [2.6–4.2] mg/kg/day, n = 7, *p* = 0.016).

In all seven patients who developed PD, PD occurred following midazolam dose reduction on that day compared with the day prior to PD detection. In six out of seven patients with PD, PD occurred following the reduction in the total midazolam concentration on the day with PD compared with the day prior to PD. In one patient, PD occurred after an increase in midazolam concentration (30%), while the dose was reduced (from 2.6 to 2.4 mg/kg/day). This patient had developed renal impairment earlier during PICU admission for which dialysis was initiated. The median (IQR) percentage difference in midazolam concentration the day with PD compared with the day prior to PD was −76.1 (−91.2–−59.8) % (n = 7). In five out of six patients who developed PD after a drop in concentration, this difference in midazolam concentration was the strongest negative change during their PICU admission. The other patient had a stronger negative change in midazolam concentration the day after PD. The patient who developed PD after an increase in the midazolam concentration had stronger fluctuating positive and negative changes in midazolam concentration on multiple days prior to the day PD was observed. Patients without PD had a less strong positive or negative percentage change in midazolam concentration between two consecutive days (median (IQR) −52.4 [−79.4–−2.4] %, *p* = 0.198) than patients with PD.

## 4. Discussion

Our observational study sought to explore the relationship between midazolam plasma concentration and the development of PD in critically ill children. This study highlights an important relationship between midazolam concentrations and dosage in relation to the development of PD during PICU admission.

We found that patients who developed PD had significantly lower midazolam concentrations on the day of PD compared to the day before PD detection. Similar findings were made when the active metabolites 1-hydroxymidazolam and 1-hydroxymidazolam glucuronide were considered. For the second endpoint, we also considered midazolam dosage in relation to the development of PD. In all patients, PD developed after midazolam dose reduction, which in almost all patients led to a decrease in midazolam concentration. Additionally, we found that patients who developed PD had significantly higher cumulative midazolam doses compared to those without PD. Patients who developed PD received a higher daily dose and/or received midazolam for a longer period of time.

This study is in line with previous studies, as previous studies have also shown that patients that require a higher cumulative dosage of benzodiazepines can be more prone to developing PD, possibly due to prolonged exposure to sedative agents [5,8]. Previous studies have found that a sudden drop in benzodiazepine dosage is related to the development of withdrawal symptoms [29]. Even though withdrawal symptoms and PD have similarities, our research has validated the development of PD with a validated SOS-PD score, which allows for a more precise differentiation between withdrawal symptoms and PD [26]. Both conditions can present with overlapping symptoms, such as agitation, altered mental status, and sleep disturbances, making differentiation challenging. However, key discriminative features include confusion, disorientation, decreased awareness, and apathy, which are less prominent in withdrawal [26]. Additionally, the SOS-PD score helps identify PD specifically by targeting symptoms that distinguish it from withdrawal, although only a few items on the scale are truly discriminative between the two conditions. This overlap underscores the complexity of managing critically ill children and highlights the need for careful clinical assessment and validated diagnostic tools. In addition, previous research indicates that midazolam concentration has no correlation with sedation, which is inconsistent with our results [30].

This study has shown that a sudden drop in the midazolam concentration could contribute to the development of PD. This suggests that PD may be triggered by abrupt changes, possibly due to rapid reductions in sedation that the brain struggles to adapt [31]. This is particularly relevant because midazolam is a benzodiazepine with a relatively short half-life, and its withdrawal, even if unintended, could precipitate neurocognitive disorders such as PD [29,32]. In clinical practice, while there were established tapering protocols for midazolam, these were not consistently followed by all healthcare providers, leading to variability in both the speed and consistency of dose reduction. Some physicians adhered to a cautious, gradual tapering approach, often reducing midazolam by 25% every 6 h in our unit, sometimes in conjunction with lorazepam. This lack of adherence to standard protocols complicated the definition of “rapid” reductions, as there was no universal practice. The fluctuations in midazolam concentrations on consecutive days was also noteworthy. Patients without PD exhibited fewer extreme fluctuations in midazolam concentrations compared to patients with PD. This finding suggests that more stable sedation management may help prevent PD. Additionally, a drop in dosage leads to lower midazolam concentrations in almost all patients. For one patient, the increase in midazolam concentration following dose reduction raises important questions about inter-individual variability in drug response and potential triggers for PD [33]. However, this patient had renal impairment, which could potentially explain the increase in midazolam concentration, as midazolam is not cleared by dialysis [34]. Furthermore, PD is a complex neuropsychiatric disorder characterised by acute changes in attention, awareness, and cognition [2]. Withdrawal symptoms often arise following prolonged use of sedatives like midazolam and are characterised by agitation, tremors, and autonomic dysregulation [35]. Differentiating between PD and withdrawal symptoms can be challenging due to overlapping clinical features, particularly in the PICU setting where patients are often sedated and mechanically ventilated [26,29]. Therefore, it might be postulated that patients also may have displayed withdrawal symptoms contributing to than rather being an actual PD because of the rapid weaning of midazolam [32].

A strength of this study is the thorough analysis of midazolam concentration and dosage in patients prior to the development of PD. Additionally, PD was assessed by specialised PICU nurses that routinely screen PD using the SOS-PD score. Our observational study has several limitations. Firstly, because this was an observational pilot study in a short period of time, we could not do any interventions and only use convenience blood samples, leading to a relatively low inclusion. Due to the reliance on convenience blood samples, we were unable to account for the potential influence of the time between the last midazolam dose administration and blood sample collection on midazolam metabolite concentrations. This variability in sample timing presents a limitation in our study, as we did not control for this factor in our analysis. Secondly, the variability in how midazolam doses were reduced presents a limitation. In clinical practice, the reduction in midazolam dosage varies between healthcare providers, which affects the speed and consistency of tapering. Some doctors opted for a cautious, gradual reduction, while others implemented more abrupt decreases. Lastly, two patients with PD were <3 months of age but the SOS-PD scale has not been validated yet for this age group [26].

This observational study signifies the need for a larger study in this patient population with confirmed PD, examining if our observations hold true as they may have clinical implications. Further research should focus on evaluating the effects of slower tapering of midazolam on the development of PD. Additionally, further research can explore the possible influence of independent risk factors such as severity of illness, narcotics, use of physical restraints, and environmental factors on the development of PD.

## 5. Conclusions

The results from our observational study suggest that a rapid drop in midazolam concentration in critically ill children admitted to the PICU may be correlated to PD. This study suggests that midazolam dosage should be reduced gradually, as rapid dosage reduction could lead to development of PD. In addition, this study highlights the importance of measuring the midazolam concentration for patients experiencing prolonged midazolam exposure to create a suitable tapering scheme of midazolam. However, we acknowledge that routine measurement of midazolam serum concentrations may not be feasible for many PICUs. Future research should focus on developing alternative strategies for tapering midazolam that are both effective and widely implementable.

## Figures and Tables

**Table 1:** Patient characteristics (n = 28). IQR = interquartile range. PRISM = Paediatric Risk of Mortality. P-RIFLE = Paediatric Risk, Injury, Failure, Loss, End Stage Renal Disease.

Characteristic	Overall Cohort (n = 28)	PD (n = 7)	No PD (n = 21)	*p*
	N (%) or Median (IQR)	N (%) or Median (IQR)	N (%) or Median (IQR)	
Gender				1
Male	14 (50.0)	4 (57.1)	10 (47.6)	
Female	14 (50.0)	3 (42.9)	11 (52.4)	
Age at admission (months)	4.0 (1.5–21.3)	16.0 (2.0–34.0)	3.0 (1.5–9.0)	
Age category				0.11
0–3 months	13 (46.4)	2 (28.6)	11 (52.3)	
≥3–24 months	9 (32.1)	2 (28.6)	7 (33.3)	0.333
≥2–5 years	3 (10.7)	2 (28.6)	1 (4.8)	
≥5–12 years	2 (7.1)	1 (14.3)	1 (4.8)	
≥ 12 years	1 (3.6)	0 (0)	1 (4.8)	
Weight (kg)	5.8 (4.4–9.1)	8.9 (4.9–14.0)	5.3 (4.2–7.7)	0.185
Height (cm)	61.3 (55.1–78.8)	74.0 (58.0–92.5)	58.0 (55.0–74.8)	0.184
BMI (kg/m^2^)	15.2 (14.2–16.3)	16.0 (14.5–18.1)	15.0 (14.2–16.3)	0.458
Admission diagnosis				0.83
Respiratory failure	13 (46.4)	3 (43.0)	10 (47.6)	
Cardiac surgery	5 (17.9)	1 (14)	4 (19.0)	
Liver failure	4 (14.3)	2 (290)	2 (9.5)	
Infectious	2 (7.1)	0 (0.0)	2 (9.5)	
Renal/metabolic disorder	2 (7.1)	0 (0.0)	2 (9.5)	
Neurologic disease	2 (7.1)	1 (14)	1 (4.8)	
Severity of illness (PRISM-III)	3.0 (2.0–5.0)	4.0 (2.0–5.0)	3.0 (1.5–4.5)	0.434
p-RIFLE admission day 3				0.529
No-Risk	20 (71.4)	4 (57.1)	16 (76.2)	
Risk	4 (14.3)	1 (14.3)	3 (14.3)	
Injury	4 (14.3)	2 (28.6)	2 (9.5)	
Failure	0 (0.0)	0 (0.0)	0 (0.0)	
p-RIFLE admission day 10				0.648
No-Risk	7 (25.0)	3 (42.9)	4 (19.1)	
Risk	2 (7.1)	0 (0.0)	2 (9.5)	
Injury	2 (7.1)	1 (14.3)	1 (4.8)	
Failure	2 (7.1)	0 (0.0)	2 (9.5)	
Need for dialysis	5 (17.9)	2 (28.6)	3 (14.3)	0.574
Type of respiratory support				0.27
None	1 (3.6)	1 (14.3)	0 (0.0)	
Conventional nasal	11 (39.3)	3 (42.9)	8 (38.1)	
Conventional oral	9 (32.1)	3 (42.9)	6 (28.6)	
Tracheostomy tube	1 (3.6)	0 (0.0)	1 (4.8)	
High-frequency oscillation	6 (21.4)	0 (0.0)	6 (28.6)	
Length of mechanical ventilation (days)	6.0 (5.0–12.5)	6.0 (4.0–13.0)	6.0 (5.0–12.0)	0.767
Length of stay PICU (days)	8.0 (6.3–28.5)	10.0 (8.0–20.0)	8.0 (6.0–33.50)	0.557
Length of stay PICU to PD (days)	8.0 (6.3–28.5)	8.0 (7.0–10.0)	8.0 (6.0–33.5)	0.979
Previous hospitalization (yes)	13 (46.4)	3 (42.9)	10 (47.6)	1
Survived to PICU discharge (yes)	26 (92.9)	6 (85.7)	20 (95.2)	0.444

**Table 2:** Co-medication.

Medicine	Overall Cohort (n = 28)	PD (n = 7)	No PD (n = 21)	*p*
	N (%) or Median (IQR)	N (%) or Median (IQR)	N (%) or Median (IQR)	
Fentanyl				
None	11 (39.3)	2 (28.6)	9 (42.9)	0.668
Yes	17 (60.7)	5 (71.4)	12 (57.1)	
Maintenance dose (mcg/kg/day)	35.4 (30.3–66.0)	37.8 (30.2–61.0)	33.6 (29.8–67.7)	0.799
Cumulative dose (mcg/kg)	189.0 (149.9–315.8)	209.5 (171.0–640.4)	178.4 (132.1–338.9)	0.383
Number of days	5.0 (3.5–6.5)	6.0 (5.5–9.5)	5.0 (3.0–6.8)	0.219
Morphine				
None	13 (46.4)	2 (28.6)	11 (52.4)	0.396
Yes	15 (53.6)	5 (71.4)	10 (47.6)	
Maintenance dose (mcg/kg/day)	210.9 (185.0–334.4)	210.9 (109.7–268.3)	246.0 (183.5–351.6)	0.594
Cumulative dose (mcg/kg)	1070.0 (710.0–2467.5)	710.0 (360.0–2539.7)	1092.5 (817.0– 2603.1)	0.371
Number of days	6.0 (3.0–9.0)	4.0 (2.5–12.5)	6.0 (3.8–7.5)	0.757
Lorazepam when tapering off midazolam				
None				
Yes	11 (39.3)	2 (28.6)	9 (42.9)	0.668
Cumulative dose (mcg/kg)	17 (60.7)	5 (71.4)	12 (57.1)	
	267.9 (132.5–684.9)	757.8 (175.6–953.3)	233.6 (113.4–455.5)	0.195
Phenobarbital				
None	20 (71.4)	5 (71.4)	15 (71.4)	1
Yes	8 (28.6)	2 (28.6)	6 (28.6)	
Fluconazole				
None	16 (57.1)	7 (100.0)	19 (90.5)	1
Yes	2 (7.1)	0 (0.0)	2 (9.5)	

## Data Availability

Data from this study is available from M.K. upon reason able request.
